# The Role of Adjuvant Chemotherapy in Metaplastic Breast Carcinoma: A Competing Risk Analysis of the SEER Database

**DOI:** 10.3389/fonc.2021.572230

**Published:** 2021-04-26

**Authors:** Tian Lan, Yunyan Lu, Ruzhen Zheng, Xiying Shao, Hua Luo, Junling He, Huifen Yang, Haibin Xu, Xiaojia Wang, Zujian Hu

**Affiliations:** ^1^Department of Breast Surgery, Hangzhou TCM Hospital Affiliated to Zhejiang Chinese Medical University, Hangzhou Hospital of Traditional Chinese Medicine, Hangzhou, China; ^2^The Second Clinical Medical College, Zhejiang Chinese Medical University, Hangzhou, China; ^3^Department of Cardiology, The First People's Hospital of Xiaoshan District, Hangzhou, China; ^4^Department of Radiotherapy, Hangzhou Cancer Hospital, Hangzhou, China; ^5^Department of Medical Oncology (Breast), Zhejiang Cancer Hospital, Cancer Hospital of the University of Chinese Academy of Sciences, Hangzhou, China

**Keywords:** chemotherapy, metaplastic breast carcinoma, nomogram, SEER, survival analysis

## Abstract

**Purpose:** Chemotherapy is the clinically recommended treatment for patients with operable metaplastic breast carcinoma (MBC); however, its impact remains controversial. This study investigated the possible role of chemotherapy in the treatment of MBC.

**Methods:** The Surveillance, Epidemiology, and End Results (SEER) database was used to identify the operable MBC patients. The competing risk analysis along with the propensity score matching (PSM) method was performed to evaluate the effect of chemotherapy. Moreover, a competing risk nomogram was built to identify prognosis in patients with MBC.

**Results:** Of the 1137 patients with MBC, 775 received chemotherapy and 362 did not receive chemotherapy. The 5-year cumulative incidence of breast cancer-specific death (BCSD) showed similar outcomes in both the Chemo and No-Chemo groups (21.1 vs. 24.3%, *p* = 0.57). Chemotherapy showed no apparent association with BCSD (HR, 1.07; 95% CI, 0.72–1.60; *p* = 0.72), even after subgroup analysis or PSM. Race, tumor size, lymph node status, and radiation were identified as the significant factors for MBC after a penalized variable selection process. In addition, a competing risk nomogram showed relatively good accuracy of prediction with a C-index of 0.766 (95% CI, 0.700–0.824).

**Conclusion:** Our findings demonstrated that chemotherapy did not improve BCSD for operable MBC patients. Thus, it may indicate the need to reduce exposure to the current chemotherapy strategies for patients with resectable MBC. Additionally, some novel treatment strategies are required urgently to identify and target the potential biomarkers.

## Introduction

Breast cancer is a common cancer worldwide and is estimated to have almost 279,100 new cases in 2020 (1). Metaplastic breast carcinoma (MBC) is a rare form of breast cancer that accounts for 1%−2% of all cases (2). The World Health Organization (WHO) has classified MBC as a heterogeneous group of tumors, including spindle cell carcinoma, squamous cell carcinoma, MBC with mesenchymal differentiation, fibromatosis-like metaplastic carcinoma, and low-grade adeno squamous carcinoma (3). The histopathologic characteristics of MBC involve the differentiation of cancer cells to the squamous epithelium or mesenchymal constituents, such as spindle, osseous, and chon droid cells (4). Most MBCs present with a triple-negative receptor status [lack the expression of estrogen receptor (ER), progesterone receptor (PR), and human epidermal growth factor receptor 2 (HER2)] (5).

Chemotherapy as a treatment strategy for MBC has shown contradictory results. MBC was found to be less chemo sensitive to the adjuvant, neoadjuvant, and palliative treatments (6–8), while other studies found that chemotherapy could improve the survival outcomes in MBC patients (5, 9, 10). The number of samples restricted most studies due to the rarity of the MBC diagnosis. The Surveillance, Epidemiology, and End Results (SEER) database that covers ~28% of the U.S. population provides a relatively large sample size for studying such a rare disease (11). Several studies with relatively large sample sizes evaluated the relationship between chemotherapy and overall survival (OS), which might not represent the actual impact of chemotherapy. It was found that the occurrence of non-breast cancer-specific death (non-BCSD) could prevent the occurrence of breast cancer-specific death (BCSD), and this could have led to result bias in these studies (12).

For these reasons, the proportional sub distribution hazards (SH) regression model and cumulative incidence function (CIF) was conducted in the SEER database to understand the effect of chemotherapy on BCSD in patients with early resectable MBC. Moreover, we tried to establish an appropriate individual assessment model based on the results of SH modeling, to help the physicians achieve more accurate and individual prognosis estimation in daily practice.

## Materials and Methods

### Study Population

Data on MBC records were obtained using the SEER 18 regions database [Incidence-SEER 18 Regs Research Data (with additional treatment fields), Nov 2018 Sub (1975-2016 varying)]. Permission to access the SEER database (ID number: 17010-Nov2018) was procured using the SEER-stat software (SEER^*^Stat 8.3.6). Therefore, this study received exemption from the Ethics Committee of Hangzhou Hospital of Traditional Chinese Medicine. Patients diagnosed before 2010 were excluded due to the lack of information on HER2 before 2010. In addition, we eliminated patients with metastasis MBC in the present study. Selection criteria to identify eligible patients were set as follows: (1) female patients; (2) aged ≥ 18 years at diagnosis; (3) histology ICD-O-3 (International Classification of Diseases for Oncology, 3rd edition) limited to MBC (8,980, 8,575, 8,572, 8,571, 8,560, 8,074, 8,072, 8,071, 8,070, and 8,052); (4) survival times ≥ 1 month. A total of 597 MBC patients with missing or incomplete clinic pathological data were excluded.

### Study Variables

The SEER database provided the following clinic pathological factors: demographic and socioeconomic data (gender, age at diagnosis, marital status, ethnicity, median household income, and insurance status); clinic pathologic features (tumor size, tumor grade, lymph node, TNM stage, ER, HER2, PR, and molecular subtype); treatment regimens; and prognostic information. The patients were divided into (a) Chemo group (patients who received chemotherapy) and (b) No-Chemo group (patients who did not receive chemotherapy) to explore the role of chemotherapy in the operable MBC. Patients were split into five groups based on their age: 18–49, 50–59, 60–69, 70–79, and ≥80 years. Ethnicity included Caucasian, African American, American Indian/Alaska Native (AI), and Asian or Pacific Islander (API). Socioeconomic status was classified into quartiles: quartile 4 (>$74,441), quartile 3 ($60,891–$74,440), quartile 2 ($52,621–$60,890), and quartile 1 (<$52,620). TNM staging was defined based on the guidelines of the 7th American Joint Committee on Cancer (AJCC), which comprised stages I–III. Tumor grade IV was combined with grade III. Treatment strategies included surgery (none, mastectomy, breast conserving surgery), radiotherapy, and chemotherapy.

### Statistical Analyses

The baseline features of patients in the Chemo group and the No-Chemo group were compared using the chi-squared test. Cumulative incidences of death (CID) for both BCSD and non-BCSD were assessed by the cumulative incidence function model. We used the SH model, a popular semi-parametric model for time-to-event data that considers competing risks, to quantify the impact of covariates on BCSD (13). The measures of prognostic effect on MBC mortality were expressed in terms of hazard ratios (HR) and 95% confidence intervals (CI).

A 1:1 propensity score matching (PSM) analysis was employed to decrease the selection bias and mimic a randomized controlled trial, as well as to re-examine the effect of chemotherapy on MBC using the R package “MatchIt” (14). We considered the standardized differences (SD) below 0.1 across the baseline variates after matching as the success of the balance (15). The critical factors identified by penalized variable selection methods, including smoothly clipped absolute deviation (SCAD), mini max concave penalty (MCP), and least absolute shrinkage and selection operator (LASSO), were adopted to construct a competing risk nomogram using the R package “regplot”.

Calibration and discrimination are two important aspects of a model validation (16). The concordance index (C-index) was assessed to evaluate the discrimination ability of the nomogram between 0.5 (no discrimination) and 1.0 (perfect discrimination). Brier score refers to the simultaneous discrimination and calibration (17). Calibration was measured graphically based on the relationship between the observed endpoints and the predictions. C-index, brier score, and calibration curves were all calculated by R package “riskRegression”. Here, all the statistical analyses were performed utilizing R (version 3.5.2, https://www.r-project.org/). A *p*-value of <0.05 was considered statistically significant.

## Results

### Patient Characteristics

In this study, 1137 patients with MBC fulfilled the eligibility criteria and were divided into (a) the Chemo group (*n* = 775; patients who received chemotherapy) and (b) the No-Chemo group (*n* = 362; patients who did not receive chemotherapy). The demographics and clinical information for these two groups are presented in Table 1. Patients in the No-Chemo group presented with higher percentage of widows (34.8 vs. 8.1%), older age (age > 70; 58.6 vs. 16.2%), higher white population (81.5 vs. 74.1%), lower histologic grade (I and II; 29.3 vs. 14.7%), higher percentage of stage I (32.6 vs. 19.7%), smaller tumor size (T0/1; 34.3 vs. 23%), more negative axillary lymph nodes (N0; 88.1 vs. 72.1%), more HER2 negative (97.8 vs. 92.6%), and less radiation (33.4 vs. 54.7%) (all *p* < 0.05) compared with those in the Chemo group. There was no remarkable difference between these groups in terms of insurance status, ER, PR, and surgery. The No-Chemo group showed an increase of non-BCSD with a growth in age (Supplementary Table 1).

**Table 1:** The descriptive characteristics of metaplastic breast cancer (MBC) before and after PSM.

	**Before matching**			**After matching**		
**Characteristic**	**No chemo**	**Chemo**	***p*-value**	**No chemo**	**Chemo**	***p*-value**
Sample size	362	775		236	236	
Marital status (%)			<0.001			0.988
Married	152 (42.0)	467 (60.3)		121 (51.3)	119 (50.4)	
Divorced	43 (11.9)	96 (12.4)		37 (15.7)	37 (15.7)	
Single	41 (11.3)	149 (19.2)		33 (14.0)	32 (13.6)	
Widowed	126 (34.8)	63 (8.1)		45 (19.1)	48 (20.3)	
Age (%)			<0.001			0.51
18–49	25 (6.9)	224 (28.9)		20 (8.5)	25 (10.6)	
50–59	59 (16.3)	226 (29.2)		63 (26.7)	55 (23.3)	
60–69	66 (18.2)	200 (25.8)		63 (26.7)	54 (22.9)	
70–79	97 (26.8)	106 (13.7)		71 (30.1)	75 (31.8)	
≥80	115 (31.8)	19 (2.5)		19 (8.1)	27 (11.4)	
Race (%)			0.019			0.735
White	295 (81.5)	574 (74.1)		194 (82.2)	187 (79.2)	
Black	42 (11.6)	145 (18.7)		32 (13.6)	34 (14.4)	
API	22 (6.1)	52 (6.7)		9 (3.8)	14 (5.9)	
AI	3 (0.8)	4 (0.5)		1 (0.4)	1 (0.4)	
Median household income (%)			0.093			0.936
Quartile 1	104 (28.7)	225 (29.0)		68 (28.8)	66 (28.0)	
Quartile 2	106 (29.3)	182 (23.5)		73 (30.9)	70 (29.7)	
Quartile 3	95 (26.2)	208 (26.8)		54 (22.9)	60 (25.4)	
Quartile 4	57 (15.7)	160 (20.6)		41 (17.4)	40 (16.9)	
Insurance (%)			1			1
Uninsured	5 (1.4)	11 (1.4)		4 (1.7)	3 (1.3)	
Insured	357 (98.6)	764 (98.6)		232 (98.3)	233 (98.7)	
Grade (%)			<0.001			0.358
I	37 (10.2)	21 (2.7)		17 (7.2)	21 (8.9)	
II	69 (19.1)	93 (12.0)		46 (19.5)	35 (14.8)	
III	256 (70.7)	661 (85.3)		173 (73.3)	180 (76.3)	
Stage (%)			<0.001			0.955
I	118 (32.6)	153 (19.7)		80 (33.9)	77 (32.6)	
II	212 (58.6)	479 (61.8)		131 (55.5)	133 (56.4)	
III	32 (8.8)	143 (18.5)		25 (10.6)	26 (11.0)	
Tumor size (%)			0.001			0.909
T0/1	124 (34.3)	178 (23.0)		84 (35.6)	82 (34.7)	
T2	166 (45.9)	403 (52.0)		109 (46.2)	113 (47.9)	
T3	55 (15.2)	135 (17.4)		34 (14.4)	30 (12.7)	
T4	17 (4.7)	59 (7.6)		9 (3.8)	11 (4.7)	
Node status (%)			<0.001			0.712
N0	319 (88.1)	559 (72.1)		205 (86.9)	199 (84.3)	
N1	31 (8.6)	153 (19.7)		18 (7.6)	25 (10.6)	
N2	9 (2.5)	45 (5.8)		9 (3.8)	9 (3.8)	
N3	3 (0.8)	18 (2.3)		4 (1.7)	3 (1.3)	
Subtype (%)			0.005			0.866
Triple negative	255 (70.4)	531 (68.5)		174 (73.7)	166 (70.3)	
HER2 enriched	6 (1.7)	36 (4.6)		3 (1.3)	4 (1.7)	
Luminal A	99 (27.3)	187 (24.1)		58 (24.6)	65 (27.5)	
Luminal B	2 (0.6)	21 (2.7)		1 (0.4)	1 (0.4)	
ER (%)			0.989			0.907
Negative	285 (78.7)	612 (79.0)		191 (80.9)	189 (80.1)	
Positive	77 (21.3)	163 (21.0)		45 (19.1)	47 (19.9)	
PR (%)			0.519			0.897
Negative	309 (85.4)	674 (87.0)		202 (85.6)	200 (84.7)	
Positive	53 (14.6)	101 (13.0)		34 (14.4)	36 (15.3)	
HER2 (%)			0.001			1
Negative	354 (97.8)	718 (92.6)		232 (98.3)	231 (97.9)	
Positive	8 (2.2)	57 (7.4)		4 (1.7)	5 (2.1)	
Surgery (%)			0.341			0.96
No surgery	10 (2.8)	22 (2.8)		7 (3.0)	6 (2.5)	
BCS	159 (43.9)	305 (39.4)		104 (44.1)	104 (44.1)	
Mastectomy	193 (53.3)	448 (57.8)		125 (53.0)	126 (53.4)	
Radiation (%)			<0.001			0.924
None/Unknown	241 (66.6)	351 (45.3)		148 (62.7)	150 (63.6)	
Yes	121 (33.4)	424 (54.7)		88 (37.3)	86 (36.4)	

*ER, estrogen; PR, progesterone; BCS, breast conserving surgery*.

A 1:1 matched cohort was obtained by performing PSM to eliminate the differences between two groups mentioned above. The demographic and clinic pathological features in this cohort were well-balanced (Table 1). All variables across the groups showed an absolute mean difference of <0.1 after PSM (Supplementary Figure 1).

### The Role of Chemotherapy in the Operable MBC

**Figure 1:**
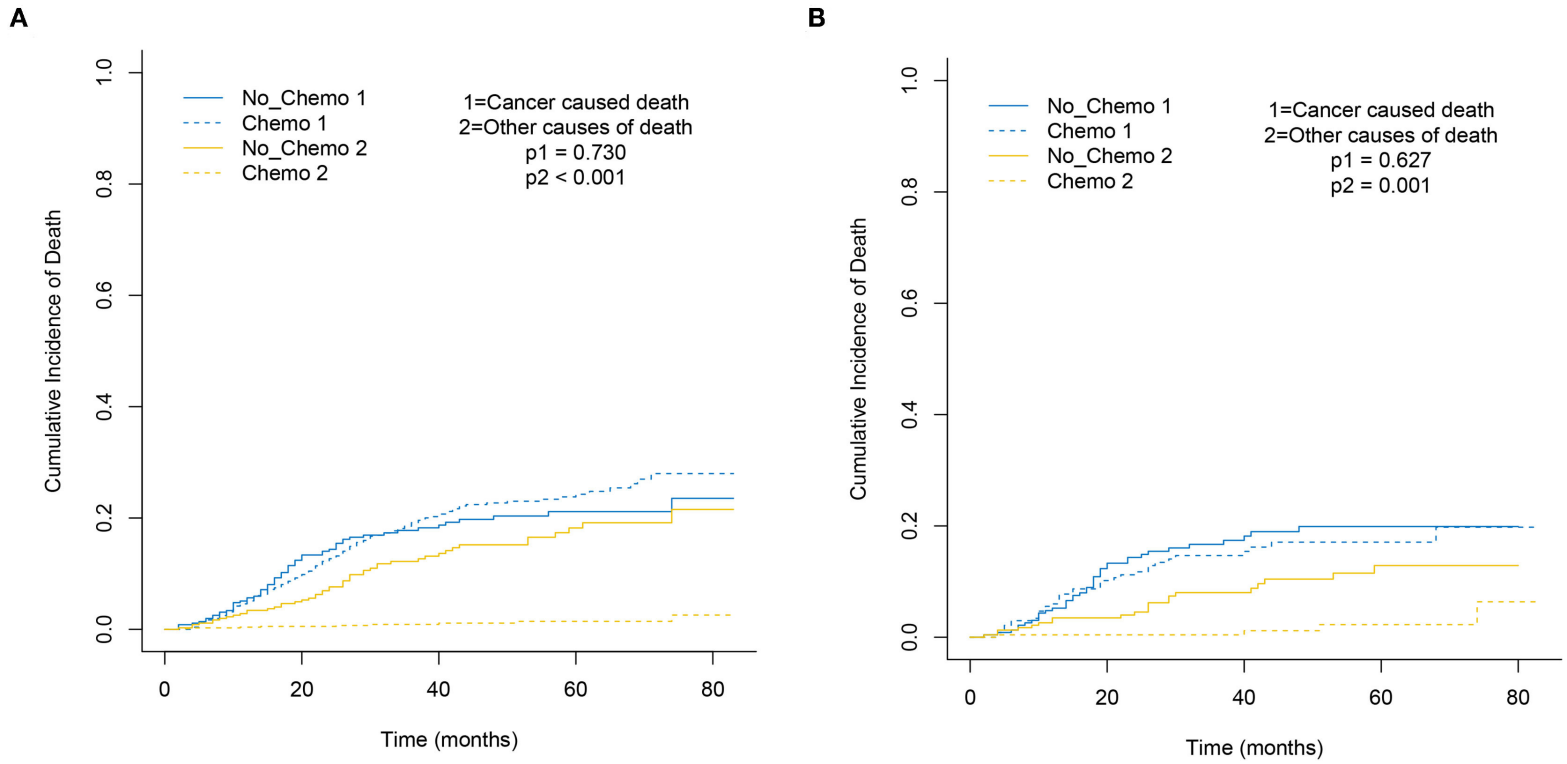
Cumulative incidence plot depicting cancer-caused death and other cause mortality based on chemotherapy before **(A)** and after **(B)** PSM.

**Figure 2:**
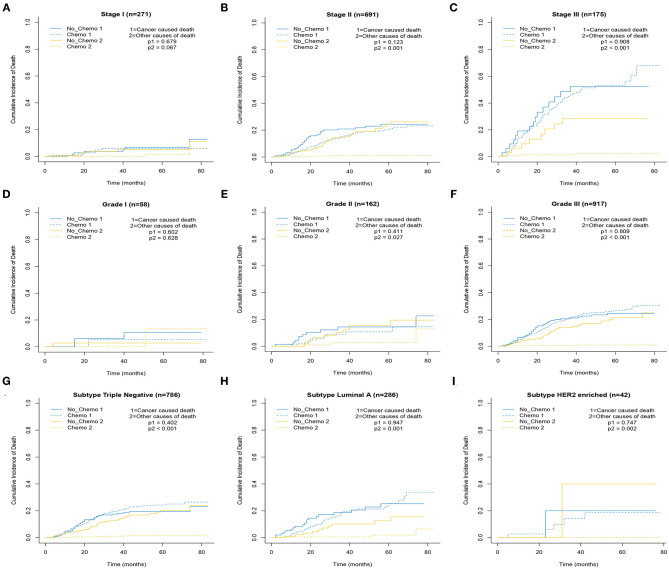
Cumulative incidence plot depicting cancer-caused death and other cause mortality according to stage **(A–C)**, grade **(D–F)**, and subtype **(G–I)**.

**Figure 3:**
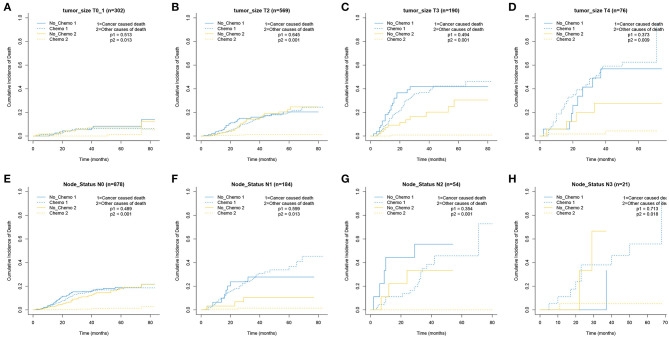
Cumulative incidence plot depicting cancer-caused death and other cause mortality according to tumor size **(A–D)** and node status **(E–H)**.

The 5-year cumulative incidence of BCSD showed similar outcomes in both the Chemo and No-Chemo groups (21.1 vs. 24.3%, *p* = 0.57) (Table 2). Additionally, the patients in the Chemo group had a lower cumulative incidence of non-BCSD than those in the No-Chemo group (1.4 vs. 18.2%, *p* < 0.001). A multivariate SH model showed that receiving chemotherapy had no apparent association with BCSD (HR, 1.07; 95% CI, 0.72–1.60; *p* = 0.72) (Figure 1A). When subgroup analysis was performed based on several clinical factors (stage, gender, subtype, tumor size, and node status), a nearly universal result was obtained for all subgroups, presenting that there was no association between chemotherapy and the 5-year cumulative incidence of BCSD (Figures 2, 3). After PSM, the new results based on the competing risk model were consistent with that of the pre-matched investigation (Figure 1B). These multidimensional results demonstrated that chemotherapy did not improve BCSD in patients with resectable MBC.

**Table 2:** The role of chemotherapy for metaplastic breast cancer (MBC) by CIF analysis and multivariate SH model before and after PSM.

		**CIF analysis**				**Multivariate SH model**		
**Status**	**Group**	**5-year CID of cancer**	***p*-value**	**5-year CID of other causes**	***p*-value**	**HR**	**95% CI**	***p*-value**
Before matching	No Chemo	0.211	0.574	0.182	<0.001	Reference	0.72–1.6	0.72
	Chemo	0.243		0.014		1.07		
After matching	No Chemo	0.199	0.627	0.129	<0.001	Reference	0.6–1.69	0.98
	Chemo	0.171		0.023		1.01		

*CID, cumulative incidences of death; HR, hazard ratio; 95% CI, 95% confidence index*.

The non-white and non-black population, single status, larger tumor size, and higher number of positive lymph nodes were risk factors associated with breast cancer-specific survival (BCSS) in the operable MBC by using the multivariate SH model (HR > 1, *p* < 0.05) (Table 3).

**Table 3:** The results of multivariate SH model for metaplastic breast cancer (MBC).

	**HR**	**95% CI**	**se (coef)**	***z***	***p*-value**
**Marital status**					
Married	Reference				
Divorced	0.93	0.57–1.51	0.249	−0.305	0.76
Single	1.61	1.13–2.31	0.183	2.604	0.01
Widowed	0.68	0.43–1.08	0.232	−1.643	0.10
**Age**					
18–49	Reference				
50–59	1.85	1.18–2.89	0.228	2.690	0.01
60–69	1.01	0.72–1.43	0.173	0.086	0.93
70–79	0.89	0.64–1.23	0.165	−0.728	0.47
≥80	1.08	0.8–1.46	0.153	0.514	0.61
**Race**					
White	Reference				
Black	1.04	0.71–1.53	0.197	0.209	0.83
API	1.61	1–2.59	0.242	1.974	0.05
AI	3.74	2.21–6.34	0.268	4.917	0.00
**Median household income**					
Quartile 1	Reference				
Quartile 2	0.75	0.56–1.01	0.151	−1.873	0.06
Quartile 3	1.11	0.84–1.48	0.146	0.742	0.46
Quartile 4	0.96	0.73–1.27	0.142	−0.256	0.80
**Insurance**					
Uninsured	Reference				
Insured	1.45	0.41–5.17	0.649	0.573	0.57
**Grade**					
I	Reference				
II	0.87	0.28–2.72	0.584	−0.246	0.81
III	1.36	0.48–3.86	0.530	0.587	0.56
**Tumor size**					
T0/1	Reference				
T2	2.16	1.25–3.75	0.281	2.748	0.01
T3	5.99	3.29–10.92	0.306	5.845	0.00
T4	7.84	4–15.34	0.343	6.008	0.00
**Node status**					
N0	Reference				
N1	1.55	1.07–2.25	0.188	2.341	0.02
N2	1.78	1.04–3.06	0.275	2.105	0.04
N3	1.99	1.08–3.66	0.310	2.221	0.03
**ER**					
Negative	Reference				
Positive	0.85	0.59–1.22	0.184	−0.896	0.37
**PR**					
Negative	Reference				
Positive	1.24	0.81–1.9	0.218	0.974	0.33
**HER2**					
Negative	Reference				
Positive	0.63	0.31–1.27	0.360	−1.296	0.20
**Surgery**					
No surgery	Reference				
BCS	0.56	0.22–1.41	0.474	−1.233	0.22
Mastectomy	0.71	0.31–1.63	0.427	−0.812	0.42
**Radiation**					
None/Unknown	Reference				
Yes	0.73	0.52–1.02	0.173	−1.838	0.07
**Chemotherapy**					
No Chemo	Reference				
Chemo	1.07	0.72–1.6	0.203	0.354	0.72

*HR, hazard ratio; 95% CI, 95% confidence index; se (coef), standard error of the regression coefficient; AI, American Indian/Alaska Native; API, Asian or Pacific Islander; ER, estrogen; PR, progesterone*.

### Construction and Validation of a Nomogram Model

**Figure 4:**
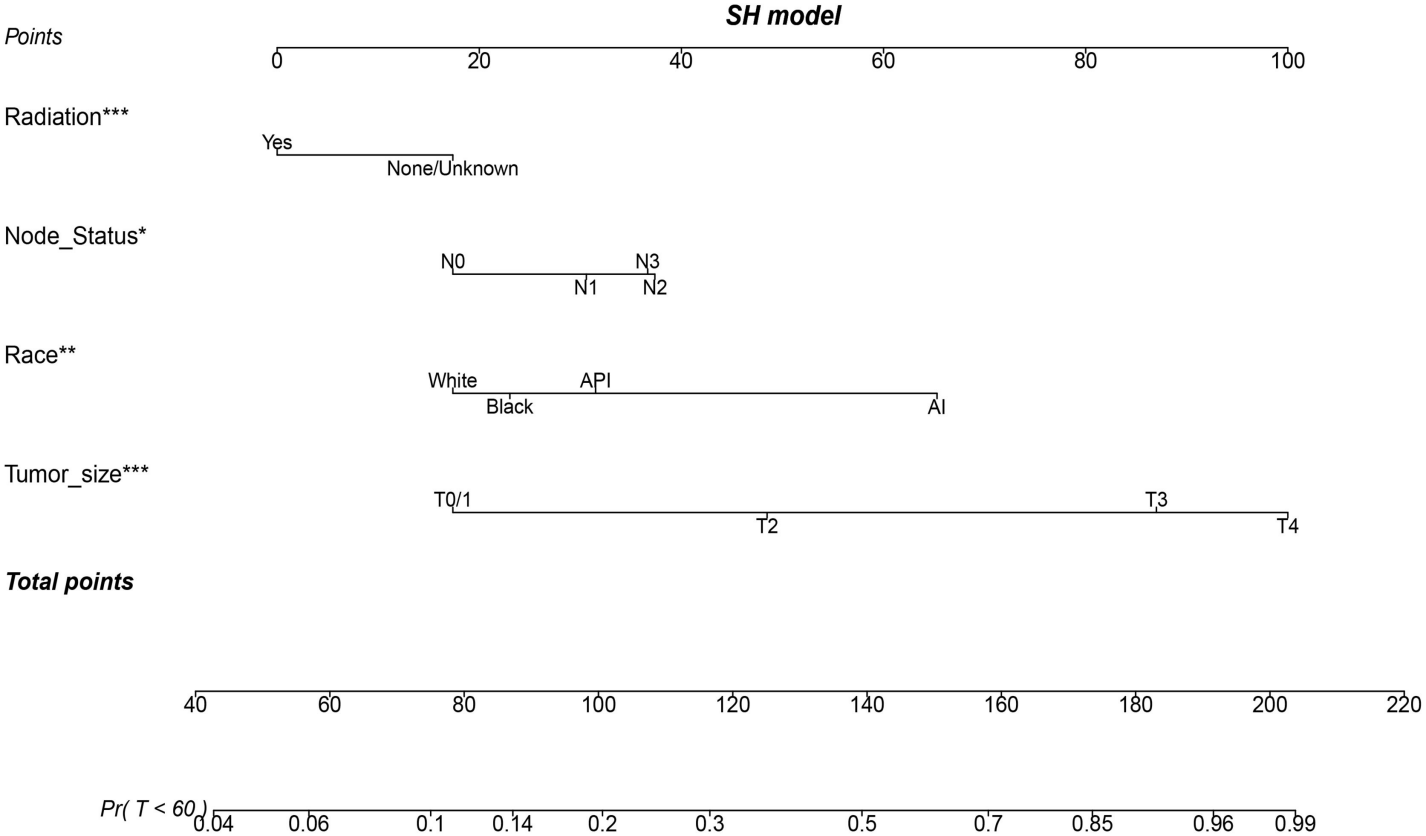
A nomogram based on SH model for predicting 60-month risk of death of patients with metaplastic breast cancer (MBC). *p < 0.05, **p < 0.01, and ***p < 0.001.

**Figure 5:**
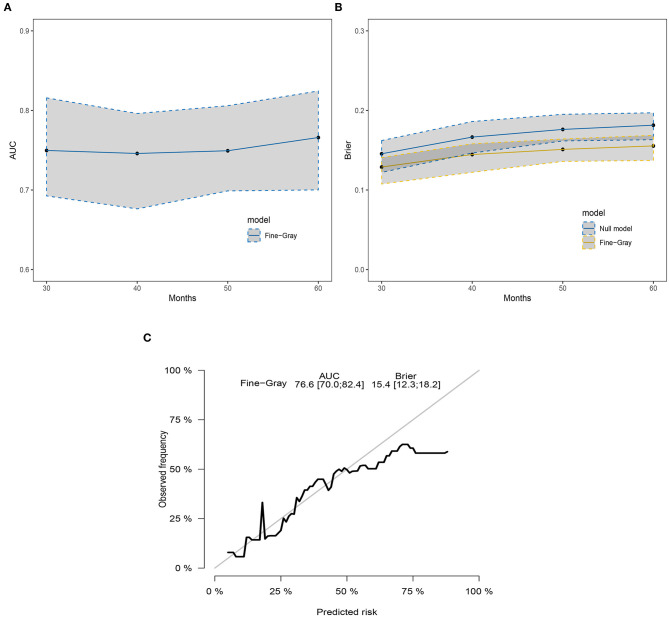
The discrimination and calibration of the SH model-based nomogram. **(A)** The time-dependent AUC graph. **(B)** The brier value graph according to the time. **(C)** The 5-year calibration plot.

Three popular methods of penalized variable selection, LASSO, SCAD, and MCP, were performed to identify the variates. Selection results and estimated coefficients are presented in Table 4. Race, tumor size, lymph node status, and radiation were identified as the significant variables. Next, we built a competing risk nomogram, together with the weighted risk score and the variables mentioned above (Figure 4). The bootstrap method was used to perform the internal validation. The nomogram showed adequate discrimination with a C-index of 0.766 (95% CI, 0.700–0.824) for a 5-year prediction (Figure 5A). Brier score was plotted over time in Figure 5B. The brier value of the nomogram we developed here was smaller than the null model, which revealed a good prediction of our model. According to the calibration plot, it was a good agreement between the prediction by nomograms and actual observations, which implied that the nomogram had good accuracy as an ideal model (Figure 5C). A total of 1033 patients were diagnosed between 2004 and 2009 in the SEER database. External validation was performed to evaluate the value of the nomogram. For 5-year BCSD, the C-index was 0.745 (95% CI, 0.714–0.801) in the testing cohort (Supplementary Figure 2A). Brier score and calibration were plotted in Supplementary Figures 2B,C.

**Table 4:** Variable selection based on estimated coefficients (SEs) by the SH model.

	**LASSO**	**SCAD**	**MCP**
Marital status	0	0	0
Age	0	0	0
Race	0.023	0.014	0.023
Median household income	0	0	0
Insurance	0	0	0
Grade	0	0	0
Tumor size	0.654	0.836	0.825
Node status	0.119	0.05	0.095
ER	0	0	0
PR	0	0	0
HER2	0	0	0
Surgery	0	0	0
Radiation	−0.007	−0.038	−0.072
Chemotherapy	0	0	0

*LASSO, least absolute shrinkage and selection operator; SCAD, smoothly clipped absolute deviation; MCP, measure correlate predict; ER, estrogen; PR, progesterone*.

## Discussion

In the time-to-event analysis, death is an event that can be observed for each patient. Several different causes can lead to death, especially in patients with breast cancer having a long survival time (18). The observation of one type of death can hinder the occurrence of another sort of death (13). However, the majority of the previous studies on MBC ignored and censored the competing risks event, which should be a key consideration in the survival analysis. Additionally, imbalances of baseline characteristics between groups can cause selection bias. PSM could reduce the selection bias and produce results similar to those from randomized control researches (19). Given the competing risks event and potential selection bias that existed, we used the PSM analysis and competing risk model to evaluate the impact of chemotherapy in the resectable MBC.

In the view of the present National Comprehensive Cancer Network guidelines, medical oncologists usually chose chemo regimens for patients with MBC based on the molecular subtypes and TNM stage in clinical practice. However, the influence of chemotherapy in this rare pathologic entity of breast cancer is controversial. In some case series, chemotherapy has shown limited efficacy in patients with MBC regardless of its administration in an adjuvant or palliative setting (20–22). Additionally, a study based on the SEER database demonstrated that chemotherapy did not lead to improved BCSS in multivariate Cox proportional hazard models (23). On the contrary, a retrospective study of the National Cancer Data Base (NCDB) indicated that administration of chemotherapy independently improved OS for patients with MBC by using multivariate analysis (10). Another population-based research has shown that chemotherapy continued to be associated with improved OS in MBC (24). Meanwhile, a report based on the SEER database concluded that chemotherapy could improve OS in MBC with triple-negative subtype by using Kaplan–Meier analysis (5). Lack of prospective random trials, different MBC patients selection, study endpoints, and statistical methods in previous retrospective studies may contribute to this controversy whether chemotherapy should be useful in the operable MBC. In the present study, chemotherapy seems to significantly decrease the incidence of non-BCSD but not BCSD. After the subgroup analysis or the PSM, the results also corroborated our finding. It demonstrated that patients in the No-Chemo group presented with older age (age > 70, 57.9 vs. 15.8%) in comparison with those in the Chemo group. Some potential comorbidities unavailable in the SEER database, older age, and a higher percentage of widowed patients in the No-Chemo group may explain the discrepancy of non-BCSD between the No-Chemo and Chemo groups partially. Therefore, we suggested that the patients with resectable MBC should be treated with less current chemotherapy or even no chemotherapy, even if it was reported that there was a worse prognosis of MBC compared with conventional triple negative breast cancer that usually triggered adjuvant chemotherapy (25).

In consideration of the ineffectiveness of current chemotherapy and poor outcomes of MBC, it is urgent to find better systemic therapy against potential molecular targets. We found that HER2 receptor was negative in major patients with MBC (94%), which was consistent with previous studies (26). Thus, anti-HER2 therapy could not be implemented as an applicable treatment option. Some comprehensive genomic studies revealed that there was a high prevalence of PI3KCA mutations and loss of PTEN in MBC, suggesting that treatments targeting the PI3K/AKT/MTOR pathway may be effective (27, 28). Some MTOR inhibitors, such as temsirolimus and everolimus, have been studied in clinical trials. They seem to suppress MBC through dual anti-cancer effects (MTOR pathway inhabitation and angiogenesis suppression) (10, 29, 30). Also, Atezolizumab plus nab-paclitaxel has been approved as the first immunotherapy regimen for patients with locally advanced or metastatic triple-negative breast tumors expressing PD-L1 (31). PD-L1 overexpression was observed in 33 of 72 (46%) MBC cases, demonstrating the potential benefit of immunotherapy for MBC (32). We found that almost 70% of MBC were triple-negative subtype, which was consistent with a previous study. It looks worthy to further investigate the effect of immunotherapy in MBC. Other studies demonstrated the abnormal activation of the canonical WNT signaling pathway by FAT1 mutations in MBC (33). MBC was also enriched with cells having stem cell-like characteristics and mesenchymal features associated with epithelial–mesenchymal transition (EMT), which may be a cause for the chemoresistant nature of MBC (34–36). Therefore, blocking the WNT signaling pathway, cancer stem cells, or EMT could be another potential novel treatment strategy.

To predict the BCSS of MBC individually, we adopted a large-scale SEER database to construct and validate a convenient nomogram, which is a valuable quantitative tool for individualized treatment. Race, tumor size, node status, and radiation were identified as the key variates to develop the first competing risk nomogram for operable MBC due to the results of SCAD, LASSO, and MCP, which may provide some clue for the treatment of MBC. Some novel therapeutic strategies are required urgently for MBC to increase the clinical application value of the nomogram.

Our study is not devoid of limitations. First, the absence of detailed information on chemotherapy in the SEER database, such as chemotherapy regimen, dose, duration, and side effect, limits the interpretation of study results. Patients that received a diagnosis amid 2010 and 2015 were recruited in the contemporary SEER database, and we speculated that most of these patients were treated with anthracyclines, taxanes, or platinum. Second, the SEER database lacks information on some crucial factors linked with prognosis, such as Ki67, targeted therapy, endocrine therapy, and comorbidities, which might cause bias. However, around 70% of patients with MBC in the current study were triple-negative MBC who did not require endocrine therapy and HER2-targeted treatment. Third, we need a larger sample size and longer follow-up duration to confirm our findings. Fourth, despite the implementation of statistical matching, some biases were inevitable due to the retrospective nature of this study. However, it is difficult to conduct prospective clinical trials due to the rarity of MBC. Thus, we would urge to interpret our findings with caution given these limitations.

In conclusion, the use of chemotherapy did not improve BCSS in the operable MBC. We suggest that a decreased necessity for current chemotherapy should be accepted to prevent overtreatment of patients with MBC. Meanwhile, some novel treatment strategies are required to target the potential biomarkers. We also constructed a competing-risk nomogram as a clinical tool to estimate the prognosis of operable MBC. Prospective studies or larger cohorts are warranted to further validate our results.

## Data Availability Statement

Publicly available datasets were analyzed in this study. This data can be found here: Surveillance, Epidemiology, and End Results (SEER) database (https://seer.cancer.gov/).

## Ethics Statement

The studies involving human participants were reviewed and approved by the Ethics Committee of Hangzhou Hospital of Traditional Chinese Medicine. Written informed consent for participation was not required for this study in accordance with the national legislation and the institutional requirements.

## Author Contributions

TL and ZH designed and managed the study. TL, YL, and RZ extracted the data. TL, YL, HY, and HL performed the analyses. TL, XS, and JH interpreted the evidence and wrote the manuscript. TL, XW, and HX revised the article. All authors contributed to the article and approved the submitted version.

## Conflict of Interest

The authors declare that the research was conducted in the absence of any commercial or financial relationships that could be construed as a potential conflict of interest.

## Supplementary Material

Supplementary Figure 1 The mean difference in all variables before and after PSM between chemotherapy and non-chemotherapy groups.

Supplementary Figure 2 The discrimination and calibration of the SH model-based nomogram in the testing cohort. **(A)** The time dependent AUC graph; **(B)** the brier value graph according to the time; **(C)** the 5-year calibration plot.

## References

[1] Siegel RL Miller KD Jemal A. Cancer statistics, 2020. CA Cancer J Clin. (2020) 70:7–30. 10.3322/caac.21590 31912902

[2] Yerushalmi R Hayes MM Gelmon KA. Breast carcinoma–rare types: review of the literature. Ann Oncol. (2009) 20:1763–70. 10.1093/annonc/mdp245 19602565

[3] Tray N Taff J Adams S. Therapeutic landscape of metaplastic breast cancer. Cancer Treat Rev. (2019) 79:101888. 10.1016/j.ctrv.2019.08.004 31491663

[4] Weigelt B Eberle C Cowell CF Ng CK Reis-Filho JS. Metaplastic breast carcinoma: more than a special type. Nat Rev Cancer. (2014) 14:147–8. 10.1038/nrc3637 25688406

[5] He X Ji J Dong R Liu H Dai X Wang C. Prognosis in different subtypes of metaplastic breast cancer: a population-based analysis. Breast Cancer Res Treat. (2019) 173:329–41. 10.1007/s10549-018-5005-6 30341462

[6] Bae SY Lee SK Koo MY Hur SM Choi MY Cho DH. The prognoses of metaplastic breast cancer patients compared to those of triple-negative breast cancer patients. Breast Cancer Res Treat. (2011) 126:471–8. 10.1007/s10549-011-1359-8 21287362

[7] Al-Hilli Z Choong G Keeney MG Visscher DW Ingle JN Goetz MP. Metaplastic breast cancer has a poor response to neoadjuvant systemic therapy. Breast Cancer Res Treat. (2019) 176:709–16. 10.1007/s10549-019-05264-2 31119569 PMC7469521

[8] Takala S Heikkila P Nevanlinna H Blomqvist C Mattson J. Metaplastic carcinoma of the breast: Prognosis and response to systemic treatment in metastatic disease. Breast J. (2019) 25:418–24. 10.1111/tbj.13234 30925636

[9] Rakha EA Tan PH Varga Z Tse GM Shaaban AM Climent F. Prognostic factors in metaplastic carcinoma of the breast: a multi-institutional study. Br J Cancer. (2015) 112:283–9. 10.1038/bjc.2014.592 25422911 PMC4453452

[10] Moreno AC Lin YH Bedrosian I Shen Y Babiera GV Shaitelman SF. Outcomes after treatment of metaplastic versus other breast cancer subtypes. J Cancer. (2020) 11:1341–50. 10.7150/jca.40817 32047541 PMC6995376

[11] Warren JL Klabunde CN Schrag D Bach PB Riley GF. Overview of the SEER-Medicare data: content, research applications, and generalizability to the United States elderly population. Med Care. (2002) 40:IV-3–18. 10.1097/00005650-200208001-00002 12187163

[12] Zhang Z Cortese G Combescure C Marshall R Lee M Lim HJ. Overview of model validation for survival regression model with competing risks using melanoma study data. Ann Transl Med. (2018) 6:325. 10.21037/atm.2018.07.38 30364028 PMC6186983

[13] Scrucca L Santucci A Aversa F. Competing risk analysis using R: an easy guide for clinicians. Bone Marrow Transplant. (2007) 40:381–7. 10.1038/sj.bmt.1705727 17563735

[14] Austin PC. An Introduction to Propensity Score Methods for Reducing the Effects of Confounding in Observational Studies. Multivariate Behav Res. (2011) 46:399–424. 10.1080/00273171.2011.568786 21818162 PMC3144483

[15] Austin PC. Balance diagnostics for comparing the distribution of baseline covariates between treatment groups in propensity-score matched samples. Stat Med. (2009) 28:3083–107. 10.1002/sim.3697 19757444 PMC3472075

[16] Steyerberg EW Vergouwe Y. Towards better clinical prediction models: seven steps for development and an ABCD for validation. Eur Heart J. (2014) 35:1925–31. 10.1093/eurheartj/ehu207 24898551 PMC4155437

[17] Gerds TA Andersen PK Kattan MW. Calibration plots for risk prediction models in the presence of competing risks. Stat Med. (2014) 33:3191–203. 10.1002/sim.6152 24668611

[18] Dai D Shi R Wang Z Zhong Y Shin VY Jin H. Competing risk analyses of medullary carcinoma of breast in comparison to infiltrating ductal carcinoma. Sci Rep. (2020) 10:560. 10.1038/s41598-019-57168-2 31953417 PMC6969020

[19] Austin PC. The use of propensity score methods with survival or time-to-event outcomes: reporting measures of effect similar to those used in randomized experiments. Stat Med. (2014) 33:1242–58. 10.1002/sim.5984 24122911 PMC4285179

[20] Akiyama F Horii R. Therapeutic strategies for breast cancer based on histological type. Breast Cancer. (2009) 16:168–72. 10.1007/s12282-009-0126-8 19479319

[21] Chen IC Lin CH Huang CS Lien HC Hsu C Kuo WH. Lack of efficacy to systemic chemotherapy for treatment of metaplastic carcinoma of the breast in the modern era. Breast Cancer Res Treat. (2011) 130:345–51. 10.1007/s10549-011-1686-9 21792625

[22] Esbah O Turkoz FP Turker I Durnali A Ekinci AS Bal O. Metaplastic breast carcinoma: case series and review of the literature. Asian Pac J Cancer Prev. (2012) 13:4645–9. 10.7314/APJCP.2012.13.9.4645 23167395

[23] Li Y Chen M Pardini B Dragomir MP Lucci A Calin GA. The role of radiotherapy in metaplastic breast cancer: a propensity score-matched analysis of the SEER database. J Transl Med. (2019) 17:318. 10.1186/s12967-019-2069-y 31547814 PMC6757394

[24] Ong CT Campbell BM Thomas SM Greenup RA Plichta JK Rosenberger LH. Metaplastic breast cancer treatment and outcomes in 2500 patients: a retrospective analysis of a national oncology database. Ann Surg Oncol. (2018) 25:2249–60. 10.1245/s10434-018-6533-3 29855830 PMC6039971

[25] El Zein D Hughes M Kumar S Peng X Oyasiji T Jabbour H. Metaplastic carcinoma of the breast is more aggressive than triple-negative breast cancer: a study from a single institution and review of literature. Clin Breast Cancer. (2017) 17:382–91. 10.1016/j.clbc.2017.04.009 28529029 PMC5537027

[26] Zhang Y Lv F Yang Y Qian X Lang R Fan Y. Clinicopathological features and prognosis of metaplastic breast carcinoma: experience of a major chinese cancer center. PLoS ONE. (2015) 10:e0131409. 10.1371/journal.pone.0131409 26115045 PMC4482719

[27] Piscuoglio S Ng CKY Geyer FC Burke KA Cowell CF Martelotto LG. Genomic and transcriptomic heterogeneity in metaplastic carcinomas of the breast. NPJ Breast Cancer. (2017) 3:48. 10.1038/s41523-017-0048-0 29214215 PMC5711926

[28] Tray N Taff J Singh B Suh J Ngo N Kwa M. Metaplastic breast cancers: Genomic profiling, mutational burden and tumor-infiltrating lymphocytes. Breast. (2019) 44:29–32. 10.1016/j.breast.2018.12.010 30609392

[29] Moroney J Fu S Moulder S Falchook G Helgason T Levenback C. Phase I study of the antiangiogenic antibody bevacizumab and the mTOR/hypoxia-inducible factor inhibitor temsirolimus combined with liposomal doxorubicin: tolerance and biological activity. Clin Cancer Res. (2012) 18:5796–805. 10.1158/1078-0432.CCR-12-1158 22927482

[30] Basho RK Yam C Gilcrease M Murthy RK Helgason T Karp DD. Comparative effectiveness of an mTOR-based systemic therapy regimen in advanced, metaplastic and nonmetaplastic triple-negative breast cancer. Oncologist. (2018) 23:1300–9. 10.1634/theoncologist.2017-0498 30139837 PMC6291334

[31] Schmid P Adams S Rugo HS Schneeweiss A Barrios CH Iwata H. Atezolizumab and nab-paclitaxel in advanced triple-negative breast cancer. N Engl J Med. (2018) 379:2108–21. 10.1056/NEJMoa1809615 30345906

[32] Joneja U Vranic S Swensen J Feldman R Chen W Kimbrough J. Comprehensive profiling of metaplastic breast carcinomas reveals frequent overexpression of programmed death-ligand 1. J Clin Pathol. (2017) 70:255–9. 10.1136/jclinpath-2016-203874 27531819 PMC5339564

[33] Ng CKY Piscuoglio S Geyer FC Burke KA Pareja F Eberle CA. The landscape of somatic genetic alterations in metaplastic breast carcinomas. Clin Cancer Res. (2017) 23:3859–70. 10.1158/1078-0432.CCR-16-2857 28153863 PMC5511565

[34] Lien HC Hsiao YH Lin YS Yao YT Juan HF Kuo WH. Molecular signatures of metaplastic carcinoma of the breast by large-scale transcriptional profiling: identification of genes potentially related to epithelial-mesenchymal transition. Oncogene. (2007) 26:7859–71. 10.1038/sj.onc.1210593 17603561

[35] Creighton CJ Li X Landis M Dixon JM Neumeister VM Sjolund A. Residual breast cancers after conventional therapy display mesenchymal as well as tumor-initiating features. Proc Natl Acad Sci USA. (2009) 106:13820–5. 10.1073/pnas.0905718106 19666588 PMC2720409

[36] Hennessy BT Gonzalez-Angulo AM Stemke-Hale K Gilcrease MZ Krishnamurthy S Lee JS. Characterization of a naturally occurring breast cancer subset enriched in epithelial-to-mesenchymal transition and stem cell characteristics. Cancer Res. (2009) 69:4116–24. 10.1158/0008-5472.CAN-08-3441 19435916 PMC2737191

