# Supplementary figures and images for: The Role of Adjuvant Chemotherapy in Metaplastic Breast Carcinoma: A Competing Risk Analysis of the SEER Database
Source: Front Oncol. 2021 Apr 26;11:572230. doi: 10.3389/fonc.2021.572230 (PMC8107469; doi:10.3389/fonc.2021.572230)

# Covariate Balance

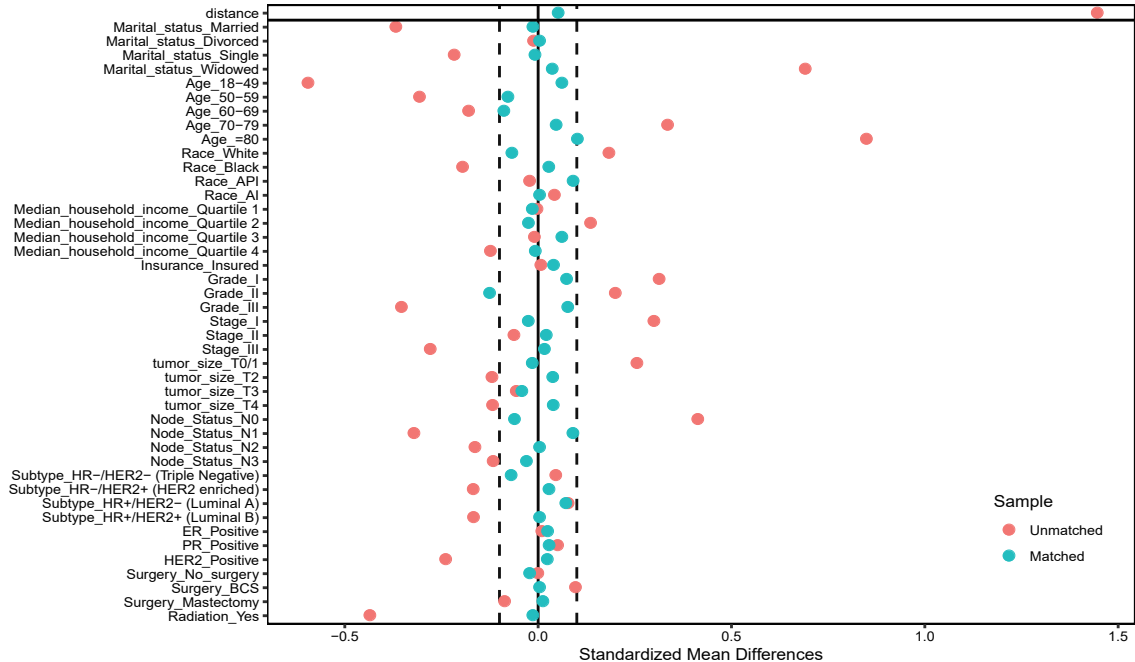

Supplement: Supplementary Figure 1 — The mean difference in all variables before and after PSM between chemotherapy and non-chemotherapy groups. [file Data_Sheet_1.PDF]

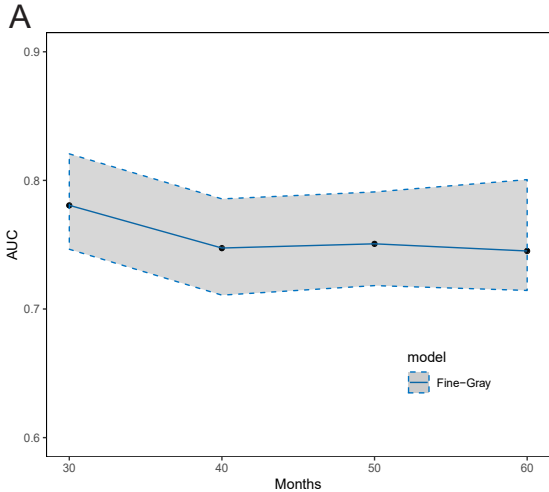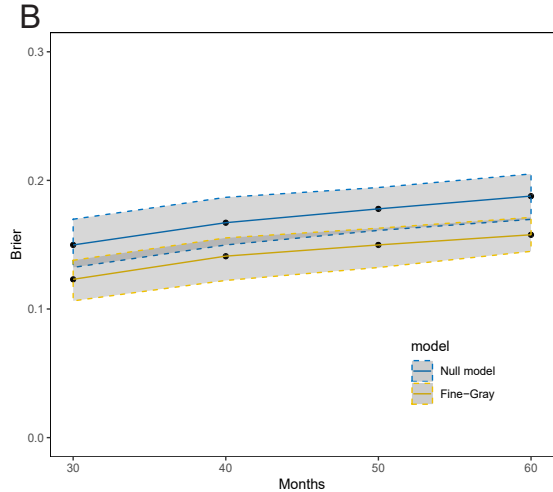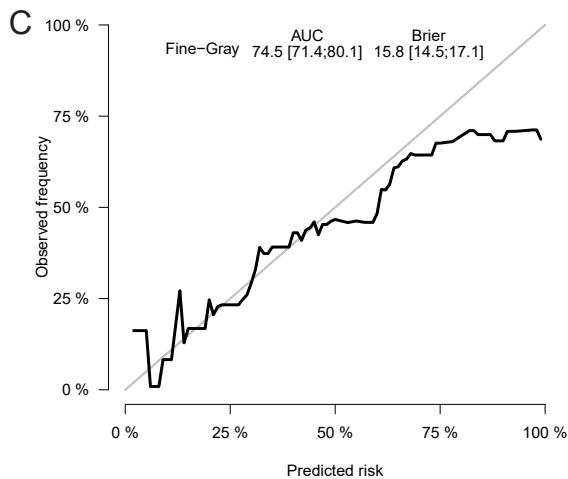

Supplement: Supplementary Figure 2 — The discrimination and calibration of the SH model-based nomogram in the testing cohort. (A) The time dependent AUC graph; (B) the brier value graph according to the time; (C) the 5-year calibration plot. [file Data_Sheet_2.PDF]
